# Functional Magnetic Resonance in the Evaluation of Oesophageal Motility Disorders

**DOI:** 10.1155/2011/367639

**Published:** 2011-08-29

**Authors:** Francesco Covotta, Luca Piretta, Danilo Badiali, Andrea Laghi, Tommaso Biondi, Enrico S. Corazziari, Valeria Panebianco

**Affiliations:** ^1^Dipartimento di Medicina Interna e Specialità Mediche, Policlinico Umberto I, Sapienza Università di Roma, 00161 Roma, Italy; ^2^Dipartimento di Scienze Radiologiche, Oncologiche e Anatomo-Patologiche, Policlinico Umberto I, Sapienza Università di Roma, 00161 Roma, Italy

## Abstract

Functional magnetic resonance imaging (fMRI) has been recently proposed for the evaluation of the esophagus. Our aim is to assess the role of fMRI as a technique to assess morphological and functional parameters of the esophagus in patients with esophageal motor disorders and in healthy controls. Subsequently, we assessed the diagnostic efficiency of fMRI in comparison to videofluoroscopic and manometric findings in the investigation of patients with esophageal motor disorders. Considering that fMRI was shown to offer valuable information on bolus transit and on the caliber of the esophagus, variations of these two parameters in the different types of esophageal motor alterations have been assessed. fMRI, compared to manometry and videofluoroscopy, showed that a deranged or absent peristalsis is significantly associated with slower transit time and with increased esophageal diameter. Although further studies are needed, fMRI represents a promising noninvasive technique for the integrated functional and morphological evaluation of esophageal motility disorders.

## 1. Introduction

The esophageal motility disorders usually present symptoms such as dysphagia or thoracic noncardiac chest pain [1].

Esophageal, and since recently high-resolution, manometry is the standard reference to diagnose these disorders by assessing the peristaltic sequences, the lower esophageal sphincter pressure, and its inhibitory reflex. However, manometry does not give information on the esophageal transit of bolus and is not valuable in case of esophageal dilatation [2]. 

Barium swallow radiology, or even better after swallow dynamic videofluoroscopy (SDV), can assess the transit of the radiopaque bolus along the esophagus and measure the luminal size of the organ. It also assesses the after swallow timely opening of the upper and lower sphincters and their coordinated activity with the wall occluding contractions, either simultaneous or propagating along the esophageal body.

Multichannel intraluminal impedance combined with manometry can offer additional information about bolus transit in the esophageal body but not on esophageal caliber [3].

Diagnosis [4–6] and followup [7] of esophageal motility disorders are usually based on manometry and SDV. 

The main limitation of SDV is the use of ionizing radiation while the magnetic resonance imaging (MRI) technique offers the same information of radiology in absence of radiation [8].

The first MRI studies began in the mid 80s [9, 10], and, currently, functional MRI (fMRI) is mostly used in cardiac area imaging for its high temporal and spatial resolution. 

In gastroenterology, MRI is used primarily in abdominal evaluation [11]. Concerning the upper gastrointestinal tract, some preliminary studies with fMRI have been performed to evaluate the oropharyngeal swallowing phase [12–14].

Manabe et al. were the first to report visualization of the esophagus and its motility in 10 patients by fMRI, introducing an optimised protocol based on T1-weighted fast-field-echo sequences [15]. 

We optimized the technique with the following imaging protocol. The examinations are performed on a 1–5 T Magnet, equipped with phased-array coil on thorax positioned. Firstly, scans are acquired with the patients in the prone position and, subsequently, are acquired in the supine position. The imaging protocol is divided into two phases. In the first phase, a breath-hold half-Fourier single-shot turbo-spin echo (HASTE) T2-weighted sequence in the axial and coronal planes is used to visualize the position of the oesophagus and its curvatures and the gastro-oesophageal junction. In the second phase, dynamic examination is performed with a single-slice slab (10 mm thickness) T1-weighted (turbo-FLASH) sequence, positioned with a median sagittal orientation on the center of the esophageal lumen, in order to depict the transit of contrast material through the esophagus. The standard parameters of the turbo-FLASH sequence are modified to obtain a temporal resolution of approximately 3-4 images/second. Immediately before the sequence starts, a small amount of contrast agent (10–15 mL) is administered directly into the oral cavity. Patients are instructed to make a single swallowing act to be synchronised with the sound signalling the beginning of the acquisition of each sequence. A suitable contrast agent for the examination should have the physical properties of barium while ensuring a valid MRI signal. Gd-DTPA-based contrast agent provides optimal signal intensity while its combination with semifluid yogurt (1 : 100) offers barium-like physical properties, improving, at the same time patient comfort and granting full compliance during the examination. The bolus is injected in the patient's mouth by the doctor or technician before the start of the dynamic sequence through a silicon catheter attached to a 20 mL syringe. Five series of dynamic acquisitions are obtained: four in the median sagittal and coronal plane to visualize esophageal motility and one on the oblique axial plane to depict lower gastroesophageal sphincter (LES) function.

**Figure 1:**
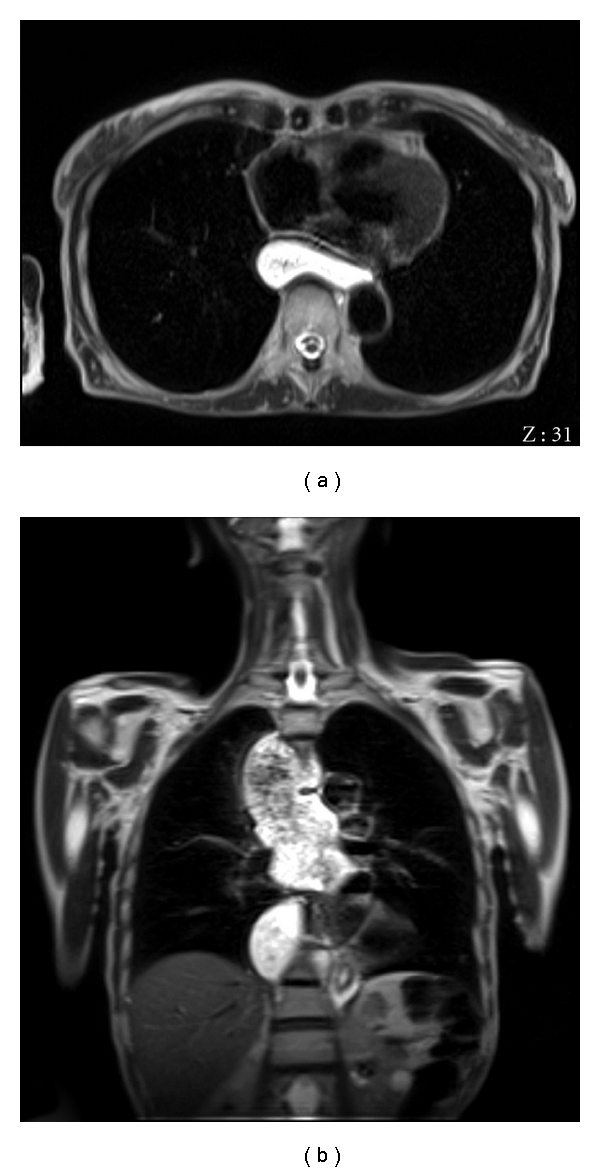
Morphologic axial (a) and coronal (b) T2w sequences demonstrate the significant increases of oesophageal caliber.

**Figure 2:**
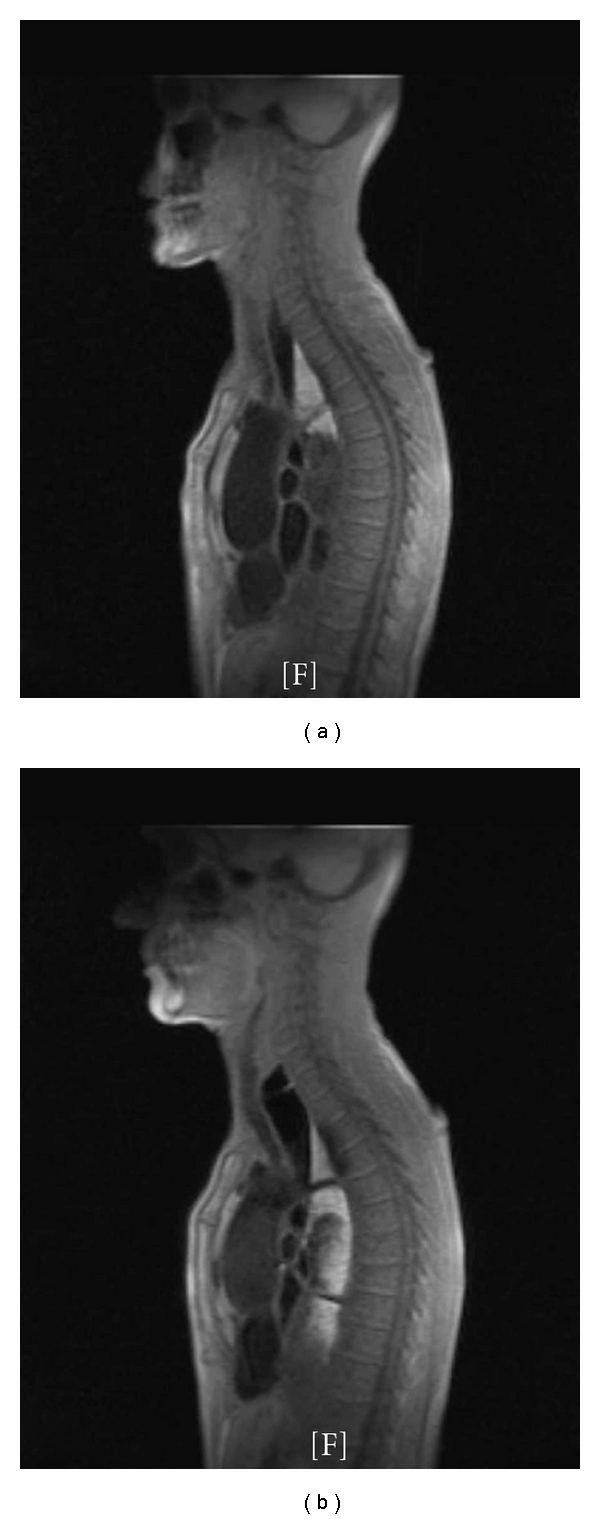
Advanced achalasia. Images acquired at 5 sec (a) and 20 sec (b) after bolus administration show distension of the oesophageal lumen (>60 mm) and replacement of the normal peristalsis by tertiary activity. The bolus does not progress into the stomach due to LES abnormalities.

In order to assess the role of fMRI as a technique to investigate the normal and the deranged motor activity of the esophagus, we have first assessed the morphological and functional aspects in 30 healthy control subjects (12 female, mean age 30 ± 7 years). In this study [8] yogurt and gadolinium were used as contrast agents. The results allowed to determine morphological and functional parameters of normality. The morphological parameters were the length of the esophagus and the esophageal calibre (Figure 1); the functional parameters were the type and the speed of propagation of esophageal wall contraction, the bolus transit time and the function, in an open or closed state, of the gastroesophageal junction (Figure 2). In addition fMRI was able to detect esophageal functional abnormalities in 7 patients with radiological and manometric diagnosis of motility disorders. 

Subsequently we have assessed the diagnostic efficiency of fMRI in comparison with SDV and manometric findings in the investigation of patients with esophageal motor disorders. In this study [16] 24 patients (14 females, mean age 55 ± 12 years) with functional esophageal dysphagia, 3 achalasic patients after pneumatic dilatation, and 15 healthy control subjects (7 female, mean age 35 ± 5 years) were investigated. fMRI correctly diagnosed achalasia in 9 patients (a dilated gastroesophageal junction and a reduction of the lumen calibre in the 3 patients after pneumatic dilatation), unspecific oesophageal body motor abnormality in 10 patients, scleroderma in 1 patient. fMRI showed a sensitivity of 87.5% and a specificity of 100% in the detection of motility alterations in comparison with manometry.

fMRI was shown to offer valuable information on bolus transit and caliber of the esophagus. In the present study we have therefore assessed how these two variables vary in the different types of esophageal motor alterations.

## 2. Patients and Methods

Twenty-four consecutive patients (14 females, age 54.6 ± 18.0 years) presenting with dysphagia and motor disorders (specific and nonspecific), diagnosed with esophageal manometry and videofluoroscopy, were enrolled. All patients had a negative upper GI endoscopy for the presence of organic disease. At the time of the study no patient had received any endoscopic or surgical treatment. fMRI was performed in all patients and 8 asymptomatic control subjects (3 females, age 27.5 ± 1.8 years) by a team of radiologists unaware of the clinical diagnosis and of the previous instrumental findings. 

At fMRI the transit time was defined by measuring the time interval between the onset of the bolus below the cricopharyngeus and its complete passage in the stomach. The caliber of the esophagus was measured as the maximal anteroposterior distance during the passage of the bolus.

Manometric diagnoses were achalasia in 10 patients, diffuse esophageal spasm in 2 patients, nutcracker esophagus in 1 patient, and nonspecific motor disorders in 11 patients. Subsequently all patients with nonspecific and specific motor disorders, except achalasic ones, were divided into groups according to the absence or presence (constant or intermittent) of peristalsis, independently from the manometric diagnosis. 

Four patients (3 of whom with hypomotility disorders or LES alterations, and 1 with nutcracker esophagus) were included in the group with constant peristalsis; 4 patients (2 patients with non-specific alterations and 2 patients with diffuse esophageal spasm) were included in the group with intermittent peristalsis and 6 patients with normal LES motor function were included in the group with absent peristalsis (Table 1). Manometric findings were then compared with the esophageal transit time and the esophageal caliber, measured by fMRI. 

## 3. Results

The control patients showed an esophageal transit time of the swallowed bolus of 8.9 sec ± 1.4 sec and a caliber of the esophagus of 13.8 ± 1.9 mm.

The esophageal transit time, measured by fMRI, increased with the gradual disappearance of peristalsis measured by esophageal manometry. In patients with esophageal motor disorders and constant peristalsis, the transit time was not different from the one of control subjects. In patients with intermittent peristalsis, the transit time doubled (17.5 ± 8.7 sec, *P* < 0.06 versus controls), and in patients with absence of peristalsis it increased up to 30.8 ± 22.9 sec (*P* < 0.06 versus controls and patients with constant peristalsis).

The achalasic patients had a transit time not significantly greater than in nonachalasic patients with aperistalsis 47.5 ± 17.2 sec, and significantly greater then controls (*P* < 0.01) and patients with constant (*P* < 0.005) and intermittent peristalsis (*P* < 0.02).

The esophageal caliber was significantly increased only in patients with absence of peristalsis, in both patients with (34.3 mm ± 15) or without (35.8 mm ± 12.4) a diagnosis of achalasia (Table 1).

Pearson correlation coefficient between oesophageal transit time (sec) versus oesophageal caliber (mm) was *R* = 0.047 (95% CI: −0.762; 1.000) in control subjects, *R* = −0.221 (95% CI: −0.964; 0.762) in patients with constant peristalsis, *R* = −0.293 (95% CI: −0.762; 0.999) in patients with intermittent peristalsis, *R* = 0.912 (95% CI: −0.999; 1.000) in nonachalasic patients with aperistalsis and *R* = 0.892 (95% CI: −1.000; 1.000) in achalasic patients.

## 4. Discussion

The comparison of the manometric and fMRI data showed an inverse relationship between the presence of manometric peristalsis and the transit time of semisolid contrast (yogurt). The bolus transit time, as it was expected, did not differ between asymptomatic controls and patients with normal peristalsis whereas it increased with intermittent peristalsis and it increased even more in the aperistaltic patients, both achalasic and non achalasic.

In addition, there is an inverse relationship between the caliber of the esophageal body observed at fMRI and the manometric assessment of peristalsis. 

In non achalasic patients with aperistalsis the dilatation of the esophagus is possibly due to primary alterations of the esophageal wall as it cannot be due to endoluminal distension secondary to content retention as it occurs in achalasia for the increased resistance at the LES. 

This study offers valuable information on the use of fMRI to assess esophageal motility by comparing its findings with those of the reference standard.

Manometry and radiology are the most widely used and invasive techniques. 

The fMRI offers integrated morphological and functional information. Non-invasive, radiation-free fMRI can be used to assess esophageal dysfunction in all patients, including pregnant women and children and in the followup of patients who require frequent controls (for example in the followup before and after pneumatic dilatation or myotomy in patients with achalasia or before and after fundoplication for gastroesophageal reflux). Differently from SDV, fMRI can also acquire multiplanar imaging, with visualisation of the esophagus and intrathoracic soft tissues in the different spatial planes, and thus detect extraluminal structural alterations, such as tumor in cases of pseudoachalasia.

The examination performed in the obligatory supine position is valuable to assess the presence of peristalsis and for the diagnosis of motility disorders. Instead caution should be applied in patients with impaired swallowing for an increased risk of aspiration. 

The analysis of fMRI data confirms its usefulness in recording transit time, esophageal size, and presence of morphological defects, but at the moment information about motility patterns are yet undefined. Manometric patterns of esophageal motor alterations allow a more defined diagnostic conclusion than fMRI. It is not possible for this technique to properly assess the extent of deranged peristalsis, the force of peristaltic contractions, and the proper time relationship between LES relaxation and esophageal peristalsis. We believe that fMRI could usefully support the interpretation of manometric findings, especially when the esophageal lumen is dilated and thus intraluminal pressure is poorly recorded, or when a morphologic anomaly is suspected in addition to the motility disorder.

 The use of contrast in fRMI may add further information about the presence of inflammation in esophageal wall and this may enable detection the presence of esophagitis, often associated with motility disorders. 

The tecnique presents, however, some limitations. fMRI is still not reliable in the diagnosis of some diseases of the wall, such as small rings, webs, and a long subtle stenosis, due to a nonoptimal spatial and temporal resolution. In order to better assess alterations of the esophageal wall profile, as seen at barium fluoroscopy, the spatial resolution during functional acquisition should be improved through the use of more adequate sequences. The temporal resolution, 2.25 frames per second (fps), is not yet comparable to the standards obtainable with X-ray (25–30 fps). To improve it and to get a true real-visualization of esophageal motility, the lowest values of flip angle, TR, and TE should be used.

Other limits are the relatively long time required to complete an fMRI examination (20–30 minutes); videofluoroscopy is usually faster (8 to 10 minutes). 

fMRI, as compared to esophageal manometry and videofluoroscopy, showed that motor alterations of the esophagus are associated with slow transit time. The transit time increases as the frequency of peristaltic activity diminishes. 

The reduced or absent peristalsis correlates significantly with the increase in size of the organ. 

In conclusion, fMRI is a promising method in the evaluation of esophageal motor disorders, as it is able to integrate functional and morphological information in a single investigation, without the limitations of invasive techniques. Further studies are needed to improve its technical limitations, but it would appear from the present study that fMRI may be useful in the study of patients with esophageal motility disorders. This new technique could be combined to standard manometry or HRM for the diagnosis and followup of the disease.

##  Conflict of Interests

The authors declare that they have no competing interests.

## Figures and Tables

**Table 1:** Esophageal transit time and size in patients and controls measured by fMRI.

Groups	*N*	Transit time (sec)	Oesophageal caliber (mm)
Controls	8	8.9 ± 1.4	13.8 ± 1.9
Motor alterations with constant peristalsis	4	8.3 ± 1.5	16.5 ± 1.3
Motor alterations with intermittent peristalsis	4	17.5 ± 8.7^∧^	16.8 ± 7.5
Aperistalsis in non achalasic patients	6	30.8 ± 22.9*	35.8 ± 12.4^§§^
Aperistalsis in achalasic patients	10	47.5 ± 17.2**	34.3 ± 15^§^

^∧^ *P* < 0.06 versus controls.

*P < 0.06 versus controls; *P* < 0.06 versus constant peristalsis.

**P < 0.01 versus controls; *P* < 0.005 versus constant peristalsis; *P* < 0.02 versus intermittent peristalsis.

^§^ *P* < 0.03 versus controls; *P* < 0.05 versus intermittent peristalsis.

^§§^ *P* < 0.06 versus controls; *P* < 0.06 versus constant peristalsis; *P* < 0.06 versus intermittent peristalsis.
